# Bacterial spectrum and proportion of multidrug-resistant bacteria in clinical samples from a regional hospital in Lambaréné, Gabon (2017–2023): Implications for antimicrobial resistance surveillance and public health

**DOI:** 10.1371/journal.pgph.0006705

**Published:** 2026-08-25

**Authors:** Maradona Daouda Agbanrin, Bayode Romeo Adegbite, Christiane Sidonie Gouleu, Sam O’neilla Oye Bingono, Jean Ulrich Muandze-Nzambe, Augustin B. Boueya, Lea Dane Damo, Lubin M. Etougou Ovono, Marina H. Biteghe Nsole, Prince Gedeon Manouana, Peter. Gottfried Kremsner, Frieder Schaumburg, Abraham Sunday Alabi, Martin P. Grobusch, Ayola Akim Adegnika, Matthew Benjamin Bransby McCall

**Affiliations:** 1 Centre de Recherches Médicales de Lambaréné (CERMEL), Lambaréné, Lambaréné, Gabon; 2 Institut für Tropenmedizin, Eberhard-Karls-Universität Tübingen, Tübingen, Germany; 3 Center of Tropical Medicine and Travel Medicine, Department of Infectious Diseases, Division of Internal Medicine, Amsterdam University Medical Centres, location University of Amsterdam, Amsterdam, The Netherlands; 4 Albert Schweitzer Hospital, Lambaréné, Gabon; 5 German Center for Infection Research (DZIF), Partner Site Tübingen, Tübingen, Germany; 6 Institute of Medical Microbiology, University Hospital Münster, Münster, Germany; 7 Health Focus GmbH., Postdam, Germany, Am Neuen Markt10, Postdam, Germany; 8 Radboud Center for Infectious Diseases, Department of Medical Microbiology, Radboud University Medical Center, Nijmegen, The Netherlands; PLOS: Public Library of Science, UNITED STATES OF AMERICA

## Abstract

This cross-sectional study investigates the spectrum and proportion of multidrug-resistant (MDR) bacteria isolated from clinical samples processed at the regional microbiology laboratory of Lambaréné, Gabon. Bacteriological results from 2017 to 2023 were retrospectively analysed to assess the rates of non-susceptibility among the most common isolates from urine, blood, stool, and swabs (wound, urethral, ear, and throat). Antimicrobial susceptibility testing and interpretation were performed according to Clinical Laboratory Standards Institute (CLSI) guidelines.A total of 5,401 cultures were reviewed, with 1,050 yielding clinically relevant bacterial isolates. The most frequently identified pathogens were *Escherichia coli* (273/1050; 26%), *Staphylococcus aureus* (192/1050; 18%), β-hemolytic *Streptococci* (160/1050; 15%), and *Klebsiella pneumoniae* (69/1050; 6.6%). Urine samples predominantly yielded *E. coli* and *K. pneumoniae*, while *S. aureus* was most common in wound and blood cultures. Overall, 23% (242/1050) of isolates were classified as MDR, with *E. coli* accounting for 40% (96/1050). The proportion of ESBL producers among Enterobacterales was 10% (55/522). Resistance to third-generation cephalosporins was notably high, especially among *K. pneumoniae* (28/69; 41%) and *E. coli* (34/273; 13%). Methicillin-resistant *S. aureus* (MRSA) represented 12% (22/192) of *S. aureus* isolates; 5.5% (58/1050) of isolates belong to the WHO critical-priority pathogen group.Clinical microbiology data from Lambaréné indicate that urinary infections are mainly associated with *E. coli*, whereas *S. aureus* predominates in wound swabs and blood cultures. The detection of WHO critical-priority pathogens in this setting supports the need for sustained microbiological monitoring and locally adapted measures to improve antibiotic use and infection prevention.

## Background

The growing spread of antimicrobial resistance (AMR) is making the treatment of bacterial infections more difficult for health systems worldwide. The emergence of multidrug-resistant (MDR) bacteria is of particular concern and a growing threat in every geographic region of the world [1]. Current estimates suggest that the burden attributable to AMR will continue to rise substantially over the coming decades, with the heaviest impact expected in Africa and South Asia [2,3]. The recent World Health Organization (WHO) Global Antimicrobial Resistance and Use Surveillance System (GLASS) report indicates that in 2023, one out of six laboratory-confirmed bacterial infections worldwide involved antibiotic-resistant bacteria, with an increase in resistance rates of *Escherichia coli* to third-generation cephalosporins and to ciprofloxacin [4]. The same report documents marked resistance in important hospital-associated pathogens such as *Klebsiella pneumoniae* and *Acinetobacter* spp.

Compared with high-income settings, low- and middle-income countries frequently report higher resistance levels across several pathogen–antibiotic combinations [5]. Beyond describing resistance patterns, an important and still insufficiently explored aspect of antimicrobial resistance research is the clinical outcomes of infections caused by multidrug-resistant (MDR) bacteria and extended-spectrum beta-lactamase (ESBL)- producing Enterobacterales, particularly in low - and middle-income countries. While resistant pathogens are often assumed to be associated with a worse prognosis, the extent to which antimicrobial resistance translates into increased disease severity, treatment failure, or mortality remains variable across settings and is poorly documented in many resource-limited contexts [6,7]. More locally generated evidence is needed to clarify how resistance patterns observed in the laboratory translate into clinical outcomes in routine care. Recent studies in different regions of Gabon show increasing rates of resistance to commonly used antibiotics in isolates from clinical specimens [8–14]. Because microbiological investigations are not consistently available, antibiotic treatment is frequently started before the causative organism and its susceptibility profile are known. In 2013, a retrospective study based on clinical samples received by the microbiology laboratory of the Centre de Recherches Médicales de Lambaréné (CERMEL), based at the Albert Schweitzer Hospital (HAS), a referral hospital in Lambaréné in the Moyen-Ogooué region of Gabon, revealed that the most commonly cultured bacteria were *Staphylococcus aureus*, *Streptococcus pyogenes, E. coli, and K. pneumoniae* [15]. More than a decade later, the present study provides updated information on pathogen distribution and resistance patterns in clinical samples from the same hospital and may help inform local interpretation of WHO priority pathogen categories and antibiotic-use policies in this setting. Therefore, our study aims to determine the bacterial spectrum and the proportion of multidrug-resistant bacteria in clinical samples collected in Lambaréné from 2017 to 2023.

## Materials and methods

### Ethics approval and consent to participate

Ethics approval for the use of data was obtained from the Institutional Ethical Review Committee (CEI/06–2023). The study adhered to the principles of the Declaration of Helsinki. The need for written informed consent was waived by the Institutional Ethical Review Committee, as this study involved anonymized retrospective data with no patient identifiers. Data extraction for research purposes started on 02/01/2024. Data were handled confidentially, with no identifiable patient information included in the analysis or reporting.

### Study design

We conducted a retrospective review of the microbiology laboratory of CERMEL, located at the referral HAS, from January 2017 to December 2023. The HAS is the regional hospital in the Moyen-Ogooué region, which provides Lambaréné and the surrounding region with many in- and out-patient services such as surgery, paediatrics, internal medicine, gynaecology, and others (e.g., dentistry, clinical laboratory diagnostics, medical microbiology). The hospital has a total of 150 beds and serves a catchment population of approximately 27000 inhabitants in the Lambaréné region [16]. The microbiology laboratory is the only one of its kind in the Moyen-Ogooué region. Clinical specimens such as urine, blood, stool, and swabs from wound, urethral, ear, and throat sent to the laboratory during the study period were included in the study. Samples originated from both hospitalised patients and outpatients.

### Sample collection and bacterial identification

Culture was done as described elsewhere [15]. Briefly, blood samples were collected into aerobic blood culture bottles for adults and children, respectively (BACTEC PLUS Aerobic and PEDS, Beckton Dickinson, Drogheda, Ireland). They were directly incubated in BD BACTEC 9050 Automated blood culture systems [17]. Samples exhibiting growth were subcultured on MacConkey agar, Chocolate agar, or Columbia blood agar (Oxoid, Basingstoke, United Kingdom) after Gram staining. Urine was sampled into sterile containers, assessed using a dipstick (Combur Test^®^, Roche, Basel, Switzerland), and cultured on Chromogenic UTI medium (Oxoid) and MacConkey agar (Oxoid).

Stool specimens were collected into sterile containers and cultured using Salmonella-Shigella agar (Oxoid), MacConkey agar (Oxoid), and Hektoen enteric agar (BD). For swabs from wounds and urethral discharge, the Gram stain morphology of principal pathogens guided the selection of appropriate media for culture. Further details of culture techniques are described elsewhere [15]. The identification of bacterial genus and species was done using a combination of colony morphology, growth characteristics on selective media, and confirmatory biochemical tests, including API strip tests (BioMérieux, Marcy‑l’Étoile, France), Streptocard latex test (BBL-BD, Heidelberg, Germany), and Pastorex Staph Plus (Bio-Rad, Hercules, California, USA).

Urine cultures yielding ≥10^5^ CFU/mL of a single uropathogen were considered indicative of significant bacteriuria. In contrast, cultures with <10³ CFU/mL were interpreted as non-significant bacteriuria, whereas samples yielding more than two distinct organisms were considered suggestive of polymicrobial bacteria. In cases where two organisms were isolated at ≥10^5^ CFU/mL, results were regarded as potentially clinically significant in specific contexts, including catheter-associated urinary tract infections and immunocompromised patients.

Only species considered clinically relevant by laboratory staff, in consultation with the treating physician, if necessary, were reported.

### Antimicrobial susceptibility testing

Antimicrobial susceptibility testing (AST) was performed using the Kirby-Bauer disc diffusion method on Muller-Hinton agar for all bacteria except for *Streptococcus* spp., *Neisseria gonorrhoeae,* and *Haemophilus influenzae*, for which blood or chocolate agar was used. Pure colonies were suspended in saline and adjusted to a turbidity equivalent of 0.5–0.6 McFarland using the VITEK DensiCHEK instrument (bioMérieux) before inoculation onto the corresponding agar plates. Antibiotic disks were then applied, and the plates were incubated overnight at 36.5–38 °C for 18–24 hours. Zone diameters were interpreted according to Clinical and Laboratory Standards Institute (CLSI) recommendations [18]. The antibiotic discs used were: penicillin (PEN, 10 units), ampicillin (AMP, 10 µg), amoxicillin-clavulanic acid (AMC, 20/10 µg), piperacillin-tazobactam (100/10 µg), cefoxitin (FOX, 30µg), cloxacillin (CLO, 5 µg), cefuroxime (CXM, 30 µg), ceftriaxone (CRO, 30 µg), ceftazidime (CAZ, 30 µg), meropenem (MEM,10 µg), chloramphenicol (CHL, 30 µg), tetracycline (TET, 30 µg), erythromycin (ERY, 15 µg), azithromycin (AZM, 15 µg), ciprofloxacin (CIP, 5 µg), gentamicin (GEN, 10 µg) and trimethoprim-sulfamethoxazole (SXT, 1.25/23.75 µg). The production of extended-spectrum beta-lactamase was confirmed in all ceftriaxone-resistant Enterobacterales using the double disc diffusion test according to the manufacturer’s instructions (Mast discs, Mast Diagnostics, Bootle, UK). This test consists of comparing the inhibition zone diameters of ceftazidime (30 μg), cefotaxime (30 μg), and cefpodoxime (10 μg) with and without clavulanic acid. All *S. aureus* strains resistant to cefoxitin were screened for PBP2a to confirm methicillin-resistant *S. aureus* (MRSA, Denka Seiken Co., LDT., Tokyo, Japan). The European Centre for Disease Control (ECDC) and the Centres for Disease Control and Prevention (CDC) definition was used to classify bacterial isolates as MDR [19]. Briefly, MDR was defined as ‘acquired non-susceptibility to at least one antimicrobial agent in ≥3 (species-relevant) antimicrobial categories.’ Extensive-drug resistance (XDR) is utilised to describe ‘non-susceptibility to at least one agent in all but ≤2 (species-relevant) antimicrobial categories’, with pan-drug resistance (PDR) described as ‘non-susceptibility to all agents in all (species-relevant) antimicrobial categories.’

### Statistical analysis

Data entered into WHONET were exported into Microsoft Excel for initial cleaning and verification before statistical analysis. WHONET [20] is a Windows-based database software package for the management of microbiology laboratory data and the analysis of antimicrobial susceptibility tests. To minimize potential selection bias, all consecutive isolates recorded during the study period were included, regardless of patient demographics or clinical setting. Duplicate specimens were identified and removed to avoid overrepresentation of strains from the same patient. Information bias was reduced by applying standardized definitions of resistance and by cross-checking data entries with original laboratory records. All cleaned data were analysed using R software, version 4.1.2. The proportions of resistant isolates were calculated by dividing the number of resistant isolates by the total number of isolates tested for each antibiotic, and the results were expressed as percentages.

## Results

### Clinical specimens and isolated pathogens

**Fig 1:**
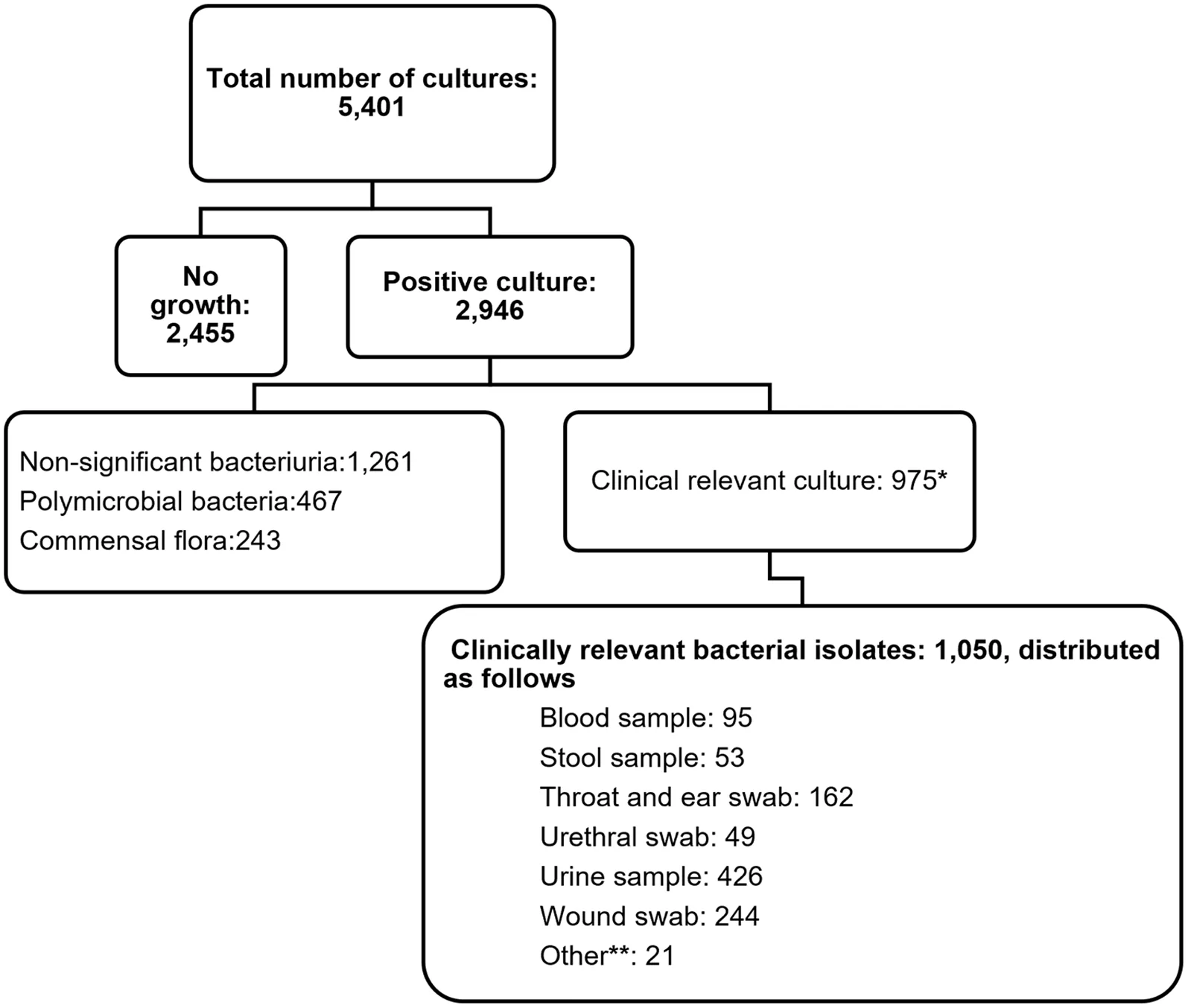
Flowchart of samples received for bacterial culture from 2017 to 2023. *Some specimens demonstrated polymicrobial growth. **Other types of samples included pleural, cerebrospinal, ascites, and synovial fluids.

A total of 5,401 laboratory result records were reviewed. Urine specimens were the most frequently processed samples (n = 3,008; 55.69%), followed by blood (n = 1,547; 28.64%), stool (n = 251; 4.65%), wound swabs (n = 378; 7%), throat swabs (n = 178; 3.30%) and urethral swabs (n = 101; 1.87%); 54.55% of samples (2,946/5,401) resulted in bacterial growth, amongst which 1,050 isolates were clinically relevant pathogens (Fig 1). The largest proportion of other samples displaying bacterial growth consisted of urine samples (n = 1,261) considered non-significant bacteriuria (< 10^3^ CFU/ml) and stool and swab samples growing commensal flora.

### Bacterial spectrum

In total, 1,050 isolates were included in this study (Fig 1). Overall, the most frequently isolated pathogens were *E. coli* (n = 273; 26%), *S. aureus* (n = 192; 18%), β-hemolytic *Streptococci* (n = 160; 15%), and *K. pneumoniae* (n = 69; 7%). The most frequently isolated pathogens from urine were *E. coli* and *K. pneumoniae*. In blood culture, the most frequent pathogenic isolates were *S*. *aureus,* followed by *K. pneumoniae* (Table 1).

**Table 1:** Top five most frequently isolated pathogens from major types of clinical samples.

Clinical Samples	Overall [n (%)]
**Urine (n = 426)**	
*E. coli*	247 (58%)
*K. pneumoniae*	46 (11%)
β-hemolytic *Streptococci*	20 (5%)
*S. aureus*	13 (3%)
Other	100 (23%)
**Blood (n = 95)**	
*S. aureus*	17 (18%)
*K. pneumoniae*	14 (15%)
*S. enterica* Typhi	13 (15%)
*E. coli*	9 (10%)
*E. cloacae*	9 (10%)
Other	33 (35%)
**Stool (n = 53)**	
*Shigella* spp.	49 (92%)
*S*. *enterica* Typhimurium	3 (5%)
*S*. *enterica* Typhi	1 (2%)
**Wound swab (n = 244)**	
*S. aureus*	123 (50%)
β-hemolytic *Streptococci*	57 (23%)
*P. aeruginosa*	12 (5%)
*E. coli*	11 (4%)
*K. pneumoniae*	4 (2%)
Other	37 (15%)

### Antibiotic non-susceptibility

**Fig 2:**
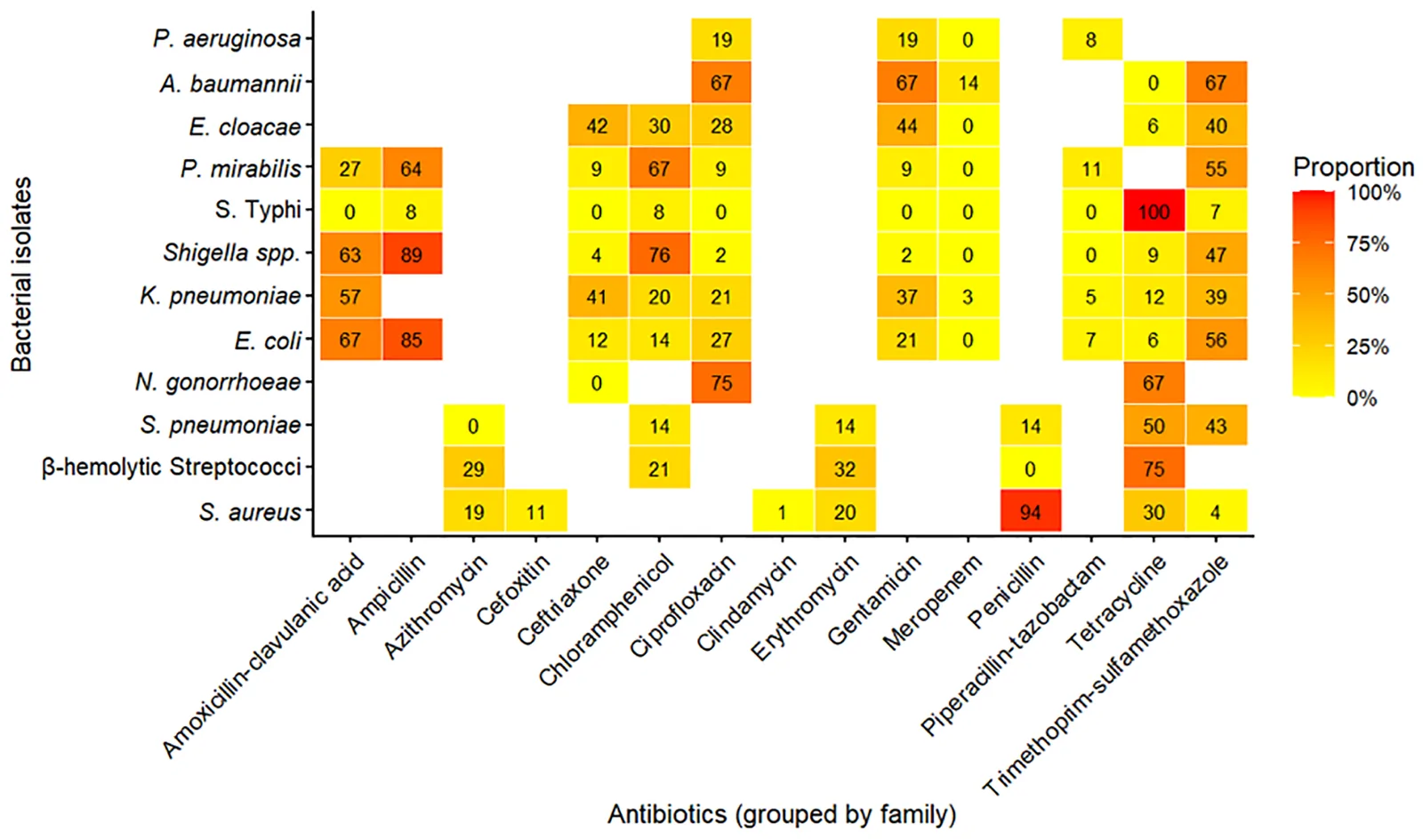
Heatmap of non-susceptibility to commonly used antibiotics amongst clinically relevant isolates. Depiction represents the proportion of isolates from each (group of) species that tested non-susceptible *in vitro* to the indicated antibiotics.

The proportion of MRSA among *S. aureus* isolates was 11% (22/192). Among the most frequently isolated Gram-negative bacteria, *E. coli* had the highest proportion of non-susceptibility to combinations of aminopenicillins with beta-lactamase inhibitors. The overall non-susceptibility against third-generation cephalosporins (ceftriaxone) was 41% (28/69) in *K*. *pneumoniae* and 12% (34/273) in *E. coli*. The proportion of ESBL producers was 27% (19/69) among *K. pneumoniae* and 8% (23/273) among *E. coli*. The proportion of ESBL producers among Enterobacterales overall was 10% (55/522). Non-susceptibility against meropenem was 3% (n = 2/69) for *K. pneumoniae* and 14% (n = 1/7) for *A*. *baumannii,* respectively (Fig 2).

### Multidrug-resistant bacteria

The overall proportion of MDR isolates was 23% (242/1,050), amongst which *E. coli* accounted for 40% (96/242). Among *E. coli* and *K. pneumoniae* isolates, 35% (96/273) and 42% (29/69), respectively, were MDR (Table 2), compared with 17% (32/192) of *S. aureus* isolates, including 22 that were MRSA*.* No XDR isolates were found. Finally, according to the WHO global priority pathogens list for antibiotic-resistant bacteria, 5.5% (58/1,050) of the isolates in our series are categorised as critical (Table 3).

**Table 2:** Distribution of MDR bacterial isolates identified in clinical samples from 2017 to 2023.

Bacterial species	Total [n (%)]	MDR [n (%)]	No MDR [n (%)]
*E. coli*	273 (26%)	96 (35%)	177 (65%)
*K. pneumoniae*	69 (6.6%)	29 (42%)	40 (58%)
*Shigella* spp.	52 (5.0%)	24 (46%)	28 (54%)
*S*. Typhi	14 (1.3%)	1 (7%)	13 (93%)
*P. mirabilis*	17 (1.6%)	4 (24%)	13 (76%)
*E. cloacae*	26 (2.5%)	13 (50%)	13 (50%)
*A. baumannii*	7 (0.7%)	3 (43%)	4 (57%)
*P. aeruginosa*	39 (3.7%)	4 (10%)	35 (90%)
*S. aureus*	192 (18.3%)	32 (17%)	160 (83%)
β-hemolytic *Streptococci*	160 (15.2%)	11 (7%)	149 (93%)
*S. pneumoniae*	9 (0.9%)	0 (0%)	9 (100%)
*N. gonorrhoeae*	32 (3.0%)	1 (3%)	31 (97%)
Other	160 (15.2%)	24 (15%)	136 (85%)
Total	1050 (100%)	242 (100%)	808 (100%)

**Table 3:** Distribution of isolates according to the WHO 2024 global priority pathogens list for antibiotic-resistant bacteria.

	Characteristic	N = 1050)
**Critical**	Enterobacterales, carbapenem-resistant	2 (0.2)
	*Acinetobacter baumannii*, Carbapenem-resistant	1 (<0.1)
	ESBL-producing Enterobacterales	55 (5)
**High priority**	*Neisseria gonorrhoeae*, fluoroquinolone-resistant	21 (2)
	*Enterococcus faecium*, vancomycin-resistant	Undetermined
	*Shigella* spp., fluoroquinolone-resistant	1 (<0.1)
	*Pseudomonas aeruginosa*, carbapenem-resistant	0
	Methicillin-resistant *Staphylococcus aureus*	22 (2)
**Medium priority**	*Streptococcus pneumoniae*, macrolide-resistant	1 (<0.1)

## Discussion

This study describes resistance patterns among bacterial pathogens isolated from routine clinical specimens processed at the CERMEL microbiology laboratory, based at HAS, between 2017 and 2023. In urine cultures, *E. coli* was the leading pathogen, followed by *K. pneumoniae*, whereas blood cultures most frequently yielded *S. aureus*, *Salmonella* Typhi, *E. coli*, and *K. pneumoniae*. This distribution is broadly consistent with earlier observations from Lambaréné [15],as well as reports from Benin [21], and many other countries across sub-Saharan Africa [21–24]. Our findings highlighted an increasing proportion (11%) of methicillin-resistant *S. aureus* (MRSA) among the *S. aureus* isolates. The proportion of methicillin-resistant *S. aureus* in our series was 11%, suggesting an increase compared with earlier data generated in the same laboratory, where MRSA represented 5% of *S. aureus* isolates [15]. Although MRSA prevalence varies widely across the continent, our findings indicate that resistant *S. aureus* remains established in this setting and should continue to be monitored. Studies from nine African countries show MRSA rates to vary between 12% and 80%, with some countries even exceeding 82% [25,26]. Among the most frequently isolated Gram-negative organisms, *E. coli* and *K. pneumoniae* showed substantial non-susceptibility to amoxicillin–clavulanic acid and ceftriaxone, whereas lower rates were observed for piperacillin–tazobactam and meropenem. Studies in Uganda also reported high resistance rates of *E*. *coli* and *K. pneumoniae* isolates to amoxicillin-clavulanic acid and ceftriaxone. Our findings of high resistance in *E*. *coli* and *K*. *pneumoniae* to amoxicillin‑clavulanic acid and ceftriaxone are consistent with Ugandan surveillance data, which reported similar trends between 2018 and 2021 [27]. The proportion of resistant *N*. *gonorrhoeae* isolates to tetracycline (67%) and to ciprofloxacin (75%) in our study, compared to previous reports from Sierra Leone (50%) [28], Nigeria (58.7–90%) [29], and Ethiopia (35–87.5%) [30], confirms alarming increasing resistance of *N*. *gonorrhoeae* to first line treatment. These findings are consistent with regional data, such as Ugandan studies that documented complete (100%) non‑susceptibility to ciprofloxacin [31]. Tetracycline and fluoroquinolones have historically been used as affordable first‑line treatments for gonococcal infections in many low‑ and middle‑income countries. Their declining efficacy will leave clinicians with fewer therapeutic choices, often forcing reliance on more expensive or less accessible agents. Together, these findings support the value of maintaining current local resistance data and using them to guide prescribing decisions and treatment recommendations. The overall proportion of MDR isolates was similar to what was reported by Cagri et al and lower than findings from Ghana (41.6) [32]. The difference can be explained by several contextual factors, including differences in sample composition, laboratory methodologies, and variation in antimicrobial prescribing practices and access to antibiotics across countries. These factors likely influence the selective pressure on bacterial populations and, consequently, the prevalence of antimicrobial resistance. Even so, the presence of MDR organisms in routine clinical samples indicates that resistance remains an important concern for both clinical care and public health. A meta-analysis performed in Ethiopia reported a pooled MDR prevalence of 72% among Klebsiella spp. isolates [33], which is substantially higher than our results. This highlights the heterogeneity of AMR burden across Africa but also underscores the common challenge of widespread resistance. The lower MDR proportion observed in this study should therefore be interpreted cautiously, particularly given the detection of ESBL-producing Enterobacterales and MRSA in this setting.

Our study and previous studies focused on ESBL-producing Enterobacterales, reporting a high proportion of carriers in Lambaréné [15,34,35]. Because *E. coli* and *K. pneumoniae* are among the main causes of urinary and bloodstream infections in this setting, their contribution to ESBL-associated resistance has direct implications for patient management. In addition, therapeutic alternatives for infections caused by these organisms may be limited in routine practice. Taken together, these observations support the need for continued laboratory-based monitoring and for antibiotic-use strategies that are informed by local microbiological data.

The AMR patterns observed in Enterobacterales reflect only one dimension of the resistance landscape in Gabon. Beyond these organisms, other high-priority pathogens also exhibit worrying resistance trends. Notably, although *Mycobacterium tuberculosis* was not assessed in our study, national data from Gabon and published by our group indicate a substantial burden of rifampicin-resistant tuberculosis: 334 RR-TB cases were identified among 3057 samples from presumptive TB patients (10.9%), and 90.7% of isolates further characterized for second-line resistance were classified as MDR-TB, underscoring the public health importance of drug-resistant tuberculosis in this setting [28–30].

### Implications for practice, policy, and antimicrobial stewardship

Our findings have several important implications for clinical practice and public health policy in Gabon. The identification of ESBL-producing Enterobacterales, MRSA, and WHO critical-priority pathogens indicates that empirical treatment may not always provide adequate coverage when microbiological confirmation is unavailable. The proportion of MDR isolates observed in this study also suggests that local antibiotic-use practices would benefit from strengthened stewardship measures, including prescribing guidance, regular review of antibiotic use, and targeted staff training. Fig 2 illustrates resistance across multiple antibiotic classes in clinically important Gram-negative pathogens, including *A. baumannii*, *K. pneumoniae*, and *E. coli*. These patterns support efforts to preserve the effectiveness of carbapenems and other key beta-lactams while identifying settings in which first-line agents may remain useful. Fig 2 illustrates the proportions of non-susceptibility to commonly prescribed antimicrobial agents among the principal clinical bacterial pathogens, highlighting heterogeneous resistance phenotypes with major implications for empirical and targeted antimicrobial therapy. *Acinetobacter baumannii* exhibits markedly elevated resistance rates to ciprofloxacin (67%) and gentamicin (67%), indicating a substantial reduction in the clinical utility of these agents against this pathogen. By comparison, *P. aeruginosa* demonstrates lower, though clinically relevant, resistance frequencies to both antibiotics (19%). Among Enterobacteriaceae, *Escherichia coli* and *K. pneumoniae* display high levels of resistance to first-line therapeutic options, particularly amoxicillin-clavulanic acid, suggesting a progressive erosion of the effectiveness of commonly used empirical regimens. Concerning Gram-positive organisms, *Staphylococcus aureus*, β-hemolytic *streptococci*, and *S. pneumoniae* remain globally susceptible to most first-line antimicrobial agents, although resistance to tetracycline reaches 30%, 75%, and 50%, respectively, underscoring species-specific variability in antimicrobial susceptibility profiles. In contrast, carbapenems and piperacillin-tazobactam retain near-complete activity and therefore represent critical reserve therapeutic options. Collectively, these findings support the necessity of tailoring antimicrobial treatment to local epidemiological and susceptibility data, while reinforcing the importance of continuous antimicrobial resistance surveillance.

At the national level, improving laboratory capacity, strengthening surveillance systems, and adapting treatment recommendations to local epidemiology may be important priorities. National efforts should prioritize investment in laboratory capacity, surveillance infrastructure, and the development of context‑specific treatment guidelines. A recent survey of physicians and nurses in Lambaréné showed gaps in knowledge about AMR, frequent empirical prescribing without microbiological confirmation, and patient‑driven requests influencing antibiotic use [30]. These behavioural and systemic factors contribute to the resistance patterns observed in our laboratory data. Several actions, including increasing health worker awareness of AMR issues and training on rational use of antibiotics, have been taken locally to reduce antibiotic overuse and antimicrobial-resistant bacteria spreading. Most of these actions were initiated in 2023; this study serves as a baseline to evaluate the efficacy of the interventions applied. From a public health perspective, the relationship between antimicrobial resistance profiles, particularly multidrug‑resistant (MDR) bacteria and ESBL‑producing Enterobacterales, and clinical outcomes remains an important area for further investigation in this setting. While this study provides robust laboratory‑based surveillance data on the distribution and resistance patterns of clinically relevant pathogens, it was not designed to explore patient‑level outcomes. Understanding the clinical and public health consequences of resistant infections, including disease severity, treatment failure, length of hospital stays, and mortality, represents a key knowledge gap. Addressing this gap will require future prospective cohort studies that integrate microbiological findings with standardized clinical outcome data. Such evidence will be essential to more accurately quantify the burden of antimicrobial resistance and to inform context‑specific antimicrobial stewardship policies, infection‑prevention strategies, and clinical guidelines in resource‑limited settings

### Strengths and limitations

One of the strengths of our study is that it reports data from several years and from the microbiology laboratory of one of the biggest referral hospitals in the Moyen Ogooué region. Resources and the supply of laboratory materials are limited in most primary health care clinics and even many hospitals in Gabon, as at many other sites in low-income countries. It is not usually possible to perform comprehensive microbiological testing. In settings where comprehensive microbiological testing cannot be performed routinely, periodic surveillance based on laboratory data may still provide practical evidence to refine empirical treatment approaches according to local pathogen distribution and resistance patterns. Our findings may also serve as a basis for future prospective studies examining prescribing practices, infection prevention, and the clinical consequences of resistant infections.

Several limitations apply to retrospective studies such as the one presented here. We were not able to distinguish community from hospital-acquired infections. The source of infection is therefore blurred. Previously, MDR bacteria were associated with healthcare-associated infections. However, some MDR bacteria are currently quite prevalent causes of community-acquired infections. Recent studies [31,36] suggest community spread of MDR bacteria, which leads to a large increase in the population at risk and subsequently an increase in the number of infections caused by MDR bacteria.

Furthermore, we did not investigate vancomycin-resistant *Enterococcus faecium* (VRE), classified as a high-priority pathogen by the WHO (2024), as our lab does not routinely perform vancomycin susceptibility testing because this antibiotic is not readily available for clinical care in our setting. This limitation may have led to an underestimation of the overall epidemiological burden of (high-priority) antimicrobial resistance.

Some samples were also taken after starting an antibiotic treatment, which may have led to selection and over-representation of resistant isolates, and for many samples, previous antibiotic intake was not reported. Due to the limited availability and high cost of commercially available blood culture bottles and regular laboratory reagent shortages, the collection of samples for microbiological culture is not systematic. Therefore, there might be a sampling bias. The clinical significance of specimen culture results could not be ascertained at an individual level, as the link between microbiology and clinical patient data could not be retrieved retrospectively. Our study did not investigate the molecular characterisation of the ESBL-producing bacteria. Finally, because antibiotic-susceptible infections will be successfully managed empirically in the community and only antibiotic-resistant infections that fail first-line empiric therapy will be (self-)referred to HAS, sampling this hospital population will likely result in an overestimation of antibiotic resistance in the community.

## Conclusion

In this study, the most frequently recovered bacterial pathogens from clinical specimens in Lambaréné included *E. coli*, *S. aureus*, and *K. pneumoniae*, with substantial levels of non-susceptibility to several antibiotics commonly used in routine care in Gabon. The occurrence of MRSA, ESBL-producing *E. coli* and *K. pneumoniae*, and MDR isolates indicates that antimicrobial resistance is already well established in this setting. Overall, these results highlight the importance of continuing laboratory surveillance in Lambaréné and making better use of microbiology results to support treatment decisions and more careful antibiotic use. Regular analysis of resistance data from commonly isolated pathogens may also help track changes over time and inform future interventions.

## Supporting information

S1 Data Datasets supporting the findings of this study. (CSV)
