# Supplementary material for: Bacterial spectrum and proportion of multidrug-resistant bacteria in clinical samples from a regional hospital in Lambaréné, Gabon (2017–2023): Implications for antimicrobial resistance surveillance and public health
Source: PLOS Glob Public Health. 2026 Aug 25;6(8):e0006705. doi: 10.1371/journal.pgph.0006705 (PMC13505894; doi:10.1371/journal.pgph.0006705)
Supplement: Supplementary file 1 [file pgph.0006705.s003.docx]

**Editor:** We noticed you have some minor occurrences of overlapping text with the following previous publication(s), which need to be addressed:

https://link.springer.com/article/10.1186/s13756-022-01053-7

In your revision, ensure you cite all your sources (including your own works), and quote or rephrase any duplicated text outside the methods section. Further consideration is dependent on these concerns being addressed

**Reply:** Thank you for this observation. We carefully revised the manuscript to remove or substantially rephrase the passages that could overlap with previously published text, including the article cited by the editor. We also ensured that statements derived from prior literature are properly attributed through citation where appropriate.

**Reviewer #1:**

1-This manuscript presents results from a retrospective and monocentric study of bacteria isolated from clinical samples processed at the regional microbiology laboratory of Lambaréné, Gabon. The authors studied the spectrum and proportion of multidrug-resistant (MDR) bacteria.In terms of structure and data presentation, this article offers a smooth read, is well-organized, and presents clear calculations. However, with only about 140 bacterial isolations per year, this sample size is far too small to achieve statistical power, meaning this work is not statistically representative of all infections in the hospital's catchment population. The critical lack of financial resources leads to non-systematic testing that is heavily biased toward problematic cases where initial therapy failed. Consequently, these data cannot yield precise local resistance tables or serve as a basis for future empirical guidelines.

**Reply**: We thank the reviewer for this important and thoughtful comment. We fully acknowledge that the relatively small number of isolates, the retrospective nature, and the non-systematic nature of microbiological testing in a resource-limited setting limit the statistical representativeness of our findings. In the revised manuscript, we have strengthened the Limitations section to explicitly acknowledge these important constraints. We have also moderated the interpretation of our findings and clarified that our results should not be considered a definitive basis for empirical treatment guidelines or precise local resistance estimates. Rather, they should be interpreted as descriptive and context-specific data that provide an initial overview of resistance patterns among clinically significant isolates processed in this setting. Despite these limitations, we believe the study remains valuable in highlighting existing diagnostic and surveillance gaps and in contributing locally generated evidence from an under-resourced context where such data remains scarce

2-Furthermore, given its high clinical relevance in the region, it is unfortunate that the authors did not encompass data regarding Mycobacterium tuberculosis in their discussion, specifically the percentage of Rifampicin-resistant strains. Ultimately, as it stands, despite the paper’s undeniable statistical flaws, these limitations must be deeply contextualized within the harsh reality of a resource-limited hospital. One cannot evaluate a study conducted in an underfunded setting through the same lens as research from a developed nation, where ample resources allow for systematic sampling and comprehensive molecular analysis. Under these challenging circumstances, the dedication of these researchers remains highly commendable, as every initiative aimed at investigating our own reality is a decisive starting point for establishing essential local antimicrobial stewardship rules, allowing us to identify current gaps and take effective action where it is most needed.

**Reply**: We thank the reviewer for this relevant comment. We agree that *Mycobacterium tuberculosis*, especially rifampicin-resistant tuberculosis (RR-TB), is highly clinically relevant in Gabon. We have now revised the Discussion to acknowledge the importance of RR-TB in Gabon and to refer to available national surveillance data, including previous work from our group showing a substantial burden of RR-TB/MDR-TB in the country. We have also clarified this point in the manuscript as a limitation and scope consideration.

Minor Comments:
1. Contextual Data: Please include the total number of hospital beds and the size of the population catchment area covered by the hospital to better understand the clinical setting.

**Reply**: We have revised the manuscript to provide additional contextual information on the hospital setting, as follows:

*The HAS is the regional hospital in the Moyen-Ogooué region, providing Lambaréné and the surrounding region with many in- and outpatient services, including surgery, paediatrics, internal medicine, gynaecology, and other services (e.g., dentistry, clinical laboratory diagnostics, medical microbiology). The hospital has a total of 150 beds and serves a catchment population of approximately 27,000 inhabitants in the Lambaréné region [16].*

2. Formatting: In the abstract, all bacterial species must be formatted in italics (e.g., Staphylococcus aureus, Escherichia coli).

**Reply**: Thank you for this observation. We carefully reviewed the abstract and the full manuscript and corrected the formatting of all bacterial species names accordingly.

3. Reference 1: Reference number 1 (line 354) cannot be opened as the link is broken. This must be corrected.

**Reply**: Thank you for identifying this issue. The reference has been corrected in the revised manuscript to ensure that the citation is accurate and accessible

4. Reference 4 & Background: Reference number 4 (line 362) should refer to the '2025 report', correct? This needs to be modified. Additionally, in the Background section (line 57), the year 2023 should be explicitly included as follows:
“The 2025 World Health Organization (WHO)’s Global Antimicrobial Resistance and Use Surveillance System (GLASS) report notes that, in 2023, one in six laboratory-….”

**Reply**: Thank you for this important correction. We revised the corresponding reference and updated the sentence in the Background section to explicitly refer to the 2025 WHO GLASS report and the year 2023, as suggested.

5. If you have access to the total number and percentage of Rifampicin-resistant Mycobacterium tuberculosis complex strains, please share them in your discussion, since Table 3 would only be truly complete with these data.

**Reply**: We thank the reviewer for this relevant comment. We agree that data on rifampicin-resistant *Mycobacterium tuberculosis* complex are of major clinical and public health importance in Gabon. Although *M. tuberculosis* was not included in our study dataset and Table 3 therefore cannot be completed with primary data from our laboratory, we have strengthened the Discussion by incorporating concrete national surveillance figures. As published by our group previously, in a nationwide retrospective analysis from Gabon, 334 rifampicin-resistant TB (RR-TB) cases were identified among 3057 samples from presumptive TB patients (approximately 10.9%), and among 281 isolates assessed for second-line resistance, 255 (90.7%) were classified as multidrug-resistant TB (MDR-TB). These data, while derived from a selected population of presumptive TB cases from routine surveillance, indicate a substantial burden of RR-TB/MDR-TB in the country and support its mention in the Discussion.

**Reviewer #2:** The manuscript meets the PLOS Global Health publication criteria. The study provides valuable and substantial data describing the increasing problem on antimicrobial resistance, specifically in Lambaréné, Gabon. The retrospective study was able to present evidence-based distributions of clinically significant bacterial isolates from commonly cultured specimens. Moreover, the study underscores the increasing proportion of MRSA among S. aureus isolates as well as the occurrence of multi-drug resistance among several groups of bacterial pathogens, which are considered critical based on the WHO 2024 global pathogen priority list. Their findings provide meaningfully local epidemiological data and further emphasize the serious threat to antimicrobial therapy. The findings in the study reiterates the importance of effective antimicrobial stewardship programs and continuous, accurate surveillance of antimicrobial resistance. The study highlighted the need for responsible and rational use of antibiotics, and the significance of AST-based antibiotic therapy. The recommendations are achievable and relevant to improving patient treatment outcomes and in mitigating the further spread and dissemination of antimicrobial resistance.

For improved clarity, consistency, and presentation of results, the authors may consider the following suggestions:

1-Re-check the format in writing all scientific names.

**Reply**: The entire manuscript has been checked meticulously for consistency and correctness of spelling of all scientific names.

2-Further describe the data extraction protocol in the Methods section, including measures taken to minimize potential selection and information bias

**Reply**: The respective paragraph has been re-written accordingly:

*Data entered into WHONET were exported into Microsoft Excel for initial cleaning and verification before statistical analysis. WHONET is a Windows-based database software package for the management of microbiology laboratory data and the analysis of antimicrobial susceptibility tests. To minimize potential selection bias, all consecutive isolates recorded during the study period were included, regardless of patient demographics or clinical setting. Duplicate specimens were identified and removed to avoid overrepresentation of strains from the same patient. Information bias was reduced by applying standardized definitions of resistance and by cross-checking data entries with original laboratory records. All cleaned data were analysed using R software, version 4.1.2. The proportions of resistant isolates were calculated by dividing the number of resistant strains by the total number of isolates tested for each antibiotic and expressed as percentages.*

3-Table 1: Title indicates “top four”, but in the wound sample, the top five isolates were enumerated.

**Reply**: Thank you for this observation. We corrected the title of Table 1 in the revised manuscript to ensure that it accurately reflects the data presented.

4-Line 40 and line 197: be consistent with the reported percentages for ESBL producers among Enterobacterales; .

**Reply:** Thank you for noting this inconsistency. We reviewed the relevant sections of the manuscript and harmonized the reported percentages for ESBL-producing Enterobacterales to ensure consistency throughout the text.

5-Maintain consistency in the presentation of data. In line 38,121, and Table 1 (p.10), data from wound swabs were presented, whereas in line 176, data from throat swabs were presented instead.

**Reply**: Thank you for identifying this inconsistency. We corrected the typographical error and harmonized the corresponding text and table so that the specimen category is presented consistently throughout the manuscript.

6-Be consistent with the terminology used throughout the manuscript. The methods section refers to “stool culture”, but data presented in Table 1 used “coproculture”.

**Reply**: Thank you for pointing out this inconsistency. We revised the terminology throughout the manuscript and now use “stool culture” consistently in both the Methods section and Table 1.

7-Provide a more comprehensive analysis and interpretation of Figure 2 that illustrates the heat map of non-susceptibility to commonly used antibiotics, specifically on the notable trends in resistance patterns of significant pathogens. This would strengthen the interpretation and clinical relevance of the findings.

**Reply**: We thank the reviewer for this valuable suggestion. In response, we expanded the Discussion to provide a more detailed interpretation of Figure 2 and to better highlight the clinical and public health relevance of the resistance patterns observed across major pathogens. Specifically, we now emphasize the high levels of non-susceptibility observed among key Gram-negative pathogens, particularly *Acinetobacter baumannii, Klebsiella pneumoniae,* and *Escherichia coli*, including reduced susceptibility to several commonly used first-line agents. For Gram-positive organisms, we refined the interpretation to reflect the generally lower resistance levels to several first-line agents while also noting important species-specific differences, such as tetracycline resistance.

Overall, these additions were intended to improve the interpretability of Figure 2 and to better connect the observed resistance patterns with antimicrobial stewardship priorities in this context.
