# Supplementary material for: Bacterial spectrum and proportion of multidrug-resistant bacteria in clinical samples from a regional hospital in Lambaréné, Gabon (2017–2023): Implications for antimicrobial resistance surveillance and public health
Source: PLOS Glob Public Health. 2026 Aug 25;6(8):e0006705. doi: 10.1371/journal.pgph.0006705 (PMC13505894; doi:10.1371/journal.pgph.0006705)
Supplement: Supplementary file 2 [file pgph.0006705.s004.docx]

**Reviewer #1**

The points raised in the previous review have been corrected. The manuscript has been improved, and the modifications have been successfully integrated.

However, during the final verification of the methodology, a critical discrepancy was identified in relation to the Antimicrobial Susceptibility Testing (AST) guidelines, the specific content of the discs, and the choice of agents tested.

1-Major Comment: Inconsistency between Guidelines and Disc Concentration
In the Materials and Methods section, the authors note that the laboratory followed CLSI guidelines for interpreting the diameters of the inhibition zones of the disc diffusion tests. However, the specific concentration reported for two antimicrobial discs is not aligned with CLSI guidelines, but rather with EUCAST guidelines:
Piperacillin-tazobactam: The authors reported the use of a 30/6 µg disc. This is the EUCAST-specific concentration. CLSI guidelines require a significantly higher concentration of 100/10 µg for this antibiotic combination.
Ceftazidime (CAZ): The authors reported using a 10 µg disc, which is in accordance with EUCAST guidelines. CLSI guidelines require a 30 µg disc for the ceftazidime test.

Implication: Because the criteria for interpreting zone diameter depend strictly on the specific disc concentration, EUCAST disc data cannot be validly interpreted using CLSI cutoff point tables and vice versa. Using the wrong table for the wrong disc concentration invalidates the clinical categorization (Susceptible/Resistant) of the isolates.

The authors should clarify which standard is used. If EUCAST discs were used, the dataset should be reanalyzed with EUCAST tables. If CLSI criteria were applied, these values are typographical errors that should be corrected to 100/10 µg and 30 µg, respectively.

**Reply :**

The antimicrobial susceptibility testing was performed using ceftazidime (30 µg) and piperacillin-tazobactam (100/10 µg) disks. Inhibition zone diameters were interpreted according to the CLSI guidelines and the corresponding clinical categories (Susceptible/Resistant). The error has now been corrected in the revised version of the manuscript.

2-Minor Comment: Omission of VRE in Study Limitations
Since vancomycin was not included in the test panel, the authors should address the epidemiological impact of this choice in the "Strengths and Limitations" section. The text needs to acknowledge that, without vancomycin data, it was impossible to determine the prevalence of vancomycin-resistant Enterococcus faecium (VRE). This data is important, as demonstrated by its position on the WHO's 2024 List of Priority Bacterial Pathogens. A brief sentence addressing this gap should be added to the limitations, and in Table 3, the value for vancomycin-resistant Enterococcus faecium should be "undetermined”. For carbapenem-resistant Pseudomonas aeruginosa, the value was zero, correct? It should be zero in the table.

**Reply :** It is indeed correct that vancomycin-resistant *E. faecium* was not investigated, as our lab does not routinely perform vancomycin susceptibility testing because vancomycin is not readily available for clinical care in our setting. The prevalence of carbapenem-resistant *P. aeruginosa* was indeed zero in our study.

Table 3 has been modified accordingly, and the following statement has been added to the Strengths and Limitations section:

“*Furthermore, we did not investigate vancomycin-resistant Enterococcus faecium (VRE), classified as a high-priority pathogen by the WHO (2024), as our lab does not routinely perform vancomycin susceptibility testing because this antibiotic is not readily available for clinical care in our setting. This limitation may have led to an underestimation of the overall epidemiological burden of (high-priority) antimicrobial resistance.*”

3-Minor Comment: Conceptual Update on Pneumococcal Resistance
There is a conceptual discrepancy regarding surveillance priorities for Streptococcus pneumoniae. Since the WHO's 2017 list of priority pathogens—and maintained in the 2024 updates—the international focus for screening high-priority pneumococci has shifted from penicillin resistance to macrolide resistance. The authors should correct Table 3.

**Reply:** We have modified Table 3 in line with the reviewer’s suggestion.

**Reviewer #2:** There were only some scientific names that were not italicized on lines 48, 201,203,295, and 299.

**Reply:** We have re-italicized the species names on lines 48, 201, 203, 295, and 299 that had inadvertently become de-italicized during preparation of the manuscript.
