# Supplementary material for: Bacterial spectrum and proportion of multidrug-resistant bacteria in clinical samples from a regional hospital in Lambaréné, Gabon (2017–2023): Implications for antimicrobial resistance surveillance and public health
Source: PLOS Glob Public Health. 2026 Aug 25;6(8):e0006705. doi: 10.1371/journal.pgph.0006705 (PMC13505894; doi:10.1371/journal.pgph.0006705)
Supplement: Peer review history

# Peer review: Decision Letter 0

**DOI:** 10.1371/journal.pgph.0006705.r001

**Authors:** Paulo Jorge Gonçalves de Bettencourt

22 May 2026

PGPH-D-26-01543

Bacterial spectrum and proportion of multidrug-resistant bacteria in clinical samples from Lambaréné, Gabon (2017–2023): implications for antimicrobial resistance surveillance and public health

PLOS Global Public Health

Dear Dr. ADEGBITE,

Thank you for submitting your manuscript to PLOS Global Public Health. After careful consideration, we feel that it has merit but does not fully meet PLOS Global Public Health’s publication criteria as it currently stands. Therefore, we invite you to submit a revised version of the manuscript that addresses the points raised during the review process.

Please submit your revised manuscript by Jun 21 2026 11:59PM. If you will need more time than this to complete your revisions, please reply to this message or contact the journal office at globalpubhealth@plos.org. When you're ready to submit your revision, log on to https://www.editorialmanager.com/pgph/ and select the 'Submissions Needing Revision' folder to locate your manuscript file.

Please include the following items when submitting your revised manuscript:

- A letter that responds to each point raised by the editor and reviewer(s). You should upload this letter as a separate file labeled 'Response to Reviewers'.
- A marked-up copy of your manuscript that highlights changes made to the original version. You should upload this as a separate file labeled 'Revised Manuscript with Track Changes'.
- An unmarked version of your revised paper without tracked changes. You should upload this as a separate file labeled 'Manuscript'.

Guidelines for resubmitting your figure files are available below the reviewer comments at the end of this letter.

As the corresponding author, your ORCID iD is verified in the submission system and will appear in the published article. PLOS supports the use of ORCID, and we encourage all coauthors to register for an ORCID iD and use it as well. Please encourage your coauthors to verify their ORCID iD within the submission system before final acceptance, as unverified ORCID iDs will not appear in the published article. *Only*  the individual author can complete the verification step; PLOS staff *cannot*  verify ORCID iDs on behalf of authors.

We look forward to receiving your revised manuscript.

Kind regards,

Paulo Jorge Gonçalves de Bettencourt, Ph.D.

Academic Editor

PLOS Global Public Health

**Journal Requirements:**

1. We noticed you have some minor occurrence of overlapping text with the following previous publication(s), which needs to be addressed:

https://link.springer.com/article/10.1186/s13756-022-01053-7

In your revision ensure you cite all your sources (including your own works), and quote or rephrase any duplicated text outside the methods section. Further consideration is dependent on these concerns being addressed.

2. Please amend your detailed Financial Disclosure statement. This is published with the article. It must therefore be completed in full sentences and contain the exact wording you wish to be published.

i. Please clarify all sources of financial support for your study. List the grants, grant numbers, and organizations that funded your study, including funding received from your institution. Please note that suppliers of material support, including research materials, should be recognized in the Acknowledgements section rather than in the Financial Disclosure.

ii. State the initials, alongside each funding source, of each author to receive each grant. For example: "This work was supported by the National Institutes of Health (####### to AM; ###### to CJ) and the National Science Foundation (###### to AM)."

iii. State what role the funders took in the study. If the funders had no role in your study, please state: “The funders had no role in study design, data collection and analysis, decision to publish, or preparation of the manuscript.”

iv. If any authors received a salary from any of your funders, please state which authors and which funders.

3. Please provide a complete Data Availability Statement in the submission form, ensuring you include all necessary access information or a reason for why you are unable to make your data freely accessible. If your research concerns only data provided within your submission, please write "All data are in the manuscript and/or supporting information files" as your Data Availability Statement.

4. Please provide separate figure files in .tif or .eps format.

For more information about figure files please see our guidelines:

https://journals.plos.org/globalpublichealth/s/figures

https://journals.plos.org/globalpublichealth/s/figures#loc-file-requirements

5. We do not publish any copyright or trademark symbols that usually accompany proprietary names, eg (R), (C), or TM  (e.g. next to drug or reagent names). Please remove all instances of trademark/copyright symbols throughout the text, including Combur Test®.

If the reviewer comments include a recommendation to cite specific previously published works, please review and evaluate these publications to determine whether they are relevant and should be cited. There is no requirement to cite these works unless the editor has indicated otherwise.

Please review your reference list to ensure that it is complete and correct. If you have cited papers that have been retracted, please include the rationale for doing so in the manuscript text, or remove these references and replace them with relevant current references. Any changes to the reference list should be mentioned in the rebuttal letter that accompanies your revised manuscript. If you need to cite a retracted article, indicate the article’s retracted status in the References list and also include a citation and full reference for the retraction notice.

**Additional Editor Comments (if provided):**

[Note: HTML markup is below. Please do not edit.]

Reviewers' comments:

Reviewer's Responses to Questions

**Comments to the Author**

1. Does this manuscript meet PLOS Global Public Health’s publication criteria?

Reviewer #1: Yes

Reviewer #2: Yes

**********

2. Has the statistical analysis been performed appropriately and rigorously?

Reviewer #1: Yes

Reviewer #2: Yes

**********

3. Have the authors made all data underlying the findings in their manuscript fully available (please refer to the Data Availability Statement at the start of the manuscript PDF file)?

The PLOS Data policy

Reviewer #1: Yes

Reviewer #2: Yes

**********

4. Is the manuscript presented in an intelligible fashion and written in standard English?

Reviewer #1: Yes

Reviewer #2: Yes

**********

**Reviewer #1:**  This manuscript presents results from a retrospective and monocentric study of bacteria isolated from clinical samples processed at the regional microbiology laboratory of Lambaréné, Gabon. The authors studied the spectrum and proportion of multidrug-resistant (MDR) bacteria.

In terms of structure and data presentation, this article offers a smooth read, is well-organized, and presents clear calculations.

However, with only about 140 bacterial isolations per year, this sample size is far too small to achieve statistical power, meaning this work is not statistically representative of all infections in the hospital's catchment population. The critical lack of financial resources leads to non-systematic testing that is heavily biased toward problematic cases where initial therapy failed. Consequently, these data cannot yield precise local resistance tables or serve as a basis for future empirical guidelines.

Furthermore, given its high clinical relevance in the region, it is unfortunate that the authors did not encompass data regarding Mycobacterium tuberculosis in their discussion, specifically the percentage of Rifampicin-resistant strains.

Ultimately, as it stands, despite the paper’s undeniable statistical flaws, these limitations must be deeply contextualized within the harsh reality of a resource-limited hospital. One cannot evaluate a study conducted in an underfunded setting through the same lens as research from a developed nation, where ample resources allow for systematic sampling and comprehensive molecular analysis. Under these challenging circumstances, the dedication of these researchers remains highly commendable, as every initiative aimed at investigating our own reality is a decisive starting point for establishing essential local antimicrobial stewardship rules, allowing to identify current gaps and take effective action where it is most needed.

Minor Comments:

1. Contextual Data: Please include the total number of hospital beds and the size of the population catchment area covered by the hospital to better understand the clinical setting.

2. Formatting: In the abstract, all bacterial species must be formatted in italics (e.g., Staphylococcus aureus, Escherichia coli).

3. Reference 1: Reference number 1 (line 354) cannot be opened as the link is broken. This must be corrected.

4. Reference 4 & Background: Reference number 4 (line 362) should refer to the '2025 report', correct? This needs to be modified. Additionally, in the Background section (line 57), the year 2023 should be explicitly included as follows:

“The 2025 World Health Organization (WHO)’s Global Antimicrobial Resistance and Use Surveillance System (GLASS) report notes that, in 2023, one in six laboratory-….”

5. If you have access to the total number and percentage of Rifampicin-resistant Mycobacterium tuberculosis complex strains, please share them in your discussion, since Table 3 would only be truly complete with these data.

**Reviewer #2:** The manuscript meets the PLOS Global Health publication criteria. The study provides valuable and substantial data describing the increasing problem on antimicrobial resistance, specifically in Lambaréné, Gabon. The retrospective study was able to present evidence-based distributions of clinically significant bacterial isolates from commonly cultured specimens. Moreover, the study underscores the increasing proportion of MRSA among S. aureus isolates as well as the occurrence of multi-drug resistance among several groups of bacterial pathogens, which are considered critical based on WHO 2024 global pathogen priority list. Their findings provide meaningfully local epidemiological data and further emphasize the serious threat to antimicrobial therapy. The findings in the study reiterates the importance of effective antimicrobial stewardship programs and continuous, accurate surveillance of antimicrobial resistance. The study highlighted the need for responsible and rational use of antibiotics, and the significance of AST-based antibiotic therapy. The recommendations are achievable and relevant to improving patient treatment outcomes and in mitigating the further spread and dissemination of antimicrobial resistance.

For improved clarity, consistency and presentation of results, the authors may consider the following suggestions:

Re-check the format in writing all scientific names.

Further describe the data extraction protocol in the Methods section, including measures taken to minimize potential selection and information bias

Table 1: Title indicates “top-four”, but in the wound sample, the top five isolates were enumerated.

line 40 and line 197: be consistent with the reported percentages for ESBL producers among Enterobacterales

Maintain consistency in the presentation of data. In line 38,121, and Table 1 (p.10), data from wound swabs were presented, whereas in line 176, data from throat swabs were presented instead.

Be consistent with terminology used throughout the manuscript. The methods section refers to “stool culture”, but data presented in Table 1 used “coproculture”.

Provide a more comprehensive analysis and interpretation of Figure 2 that illustrates the heat map of non-susceptibility to commonly used antibiotics, specifically on the notable trends in resistance patterns of significant pathogens. This would strengthen the interpretation and clinical relevance of the findings.

**********

what does this mean?). If published, this will include your full peer review and any attached files.

**Do you want your identity to be public for this peer review?**  If you choose “no”, your identity will remain anonymous but your review may still be made public.

For information about this choice, including consent withdrawal, please see our Privacy Policy

Reviewer #1: **Yes:** Clara Portugal

Reviewer #2: **Yes:** Merlyn Cruz

**********

[NOTE: If reviewer comments were submitted as an attachment file, they will be attached to this email and accessible via the submission site. Please log into your account, locate the manuscript record, and check for the action link "View Attachments". If this link does not appear, there are no attachment files.]

**Figure Resubmissions:**

# Author comment: Author response to Decision Letter 1

**DOI:** 10.1371/journal.pgph.0006705.r002

4 Jun 2026

Attachment Submitted filename: Point by point reply.docx

# Peer review: Decision Letter 1

**DOI:** 10.1371/journal.pgph.0006705.r003

**Authors:** Jen Edwards

1 Jul 2026

PGPH-D-26-01543R1

Bacterial spectrum and proportion of multidrug-resistant bacteria in clinical samples from Lambaréné, Gabon (2017–2023): implications for antimicrobial resistance surveillance and public health

PLOS Global Public Health

Dear Dr. ADEGBITE,

Thank you for submitting your manuscript to PLOS Global Public Health. After careful consideration, we feel that it has merit but does not fully meet PLOS Global Public Health’s publication criteria as it currently stands. Therefore, we invite you to submit a revised version of the manuscript that addresses the points raised during the review process.

The manuscript has been evaluated by the previous two reviewers and their comments are available below. The reviewers have raised a number of concerns that need attention. They request further clarification of the methodology in line with CLSI and EUCAST guidance, as well as discussion of the study limitations and surveillance priorities. Could you please revise the manuscript to carefully address the concerns raised?

Please submit your revised manuscript by Aug 30 2026 11:59PM. If you will need more time than this to complete your revisions, please reply to this message or contact the journal office at globalpubhealth@plos.org. When you're ready to submit your revision, log on to https://www.editorialmanager.com/pgph/ and select the 'Submissions Needing Revision' folder to locate your manuscript file.

Please include the following items when submitting your revised manuscript:

- A letter that responds to each point raised by the editor and reviewer(s). You should upload this letter as a separate file labeled 'Response to Reviewers'.
- A marked-up copy of your manuscript that highlights changes made to the original version. You should upload this as a separate file labeled 'Revised Manuscript with Track Changes'.
- An unmarked version of your revised paper without tracked changes. You should upload this as a separate file labeled 'Manuscript'.

Guidelines for resubmitting your figure files are available below the reviewer comments at the end of this letter.

As the corresponding author, your ORCID iD is verified in the submission system and will appear in the published article. PLOS supports the use of ORCID, and we encourage all coauthors to register for an ORCID iD and use it as well. Please encourage your coauthors to verify their ORCID iD within the submission system before final acceptance, as unverified ORCID iDs will not appear in the published article. *Only*  the individual author can complete the verification step; PLOS staff *cannot*  verify ORCID iDs on behalf of authors.

We look forward to receiving your revised manuscript.

Kind regards,

Jen Edwards

Staff Editor

PLOS Global Public Health

**Journal Requirements:**

If the reviewer comments include a recommendation to cite specific previously published works, please review and evaluate these publications to determine whether they are relevant and should be cited. There is no requirement to cite these works unless the editor has indicated otherwise.

**Additional Editor Comments (if provided):**

[Note: HTML markup is below. Please do not edit.]

Reviewers' comments:

Reviewer's Responses to Questions

**Comments to the Author**

Reviewer #1: All comments have been addressed

Reviewer #2: (No Response)

**********

publication criteria?

Reviewer #1: Partly

Reviewer #2: Yes

**********

3. Has the statistical analysis been performed appropriately and rigorously?

Reviewer #1: Yes

Reviewer #2: Yes

**********

4. Have the authors made all data underlying the findings in their manuscript fully available (please refer to the Data Availability Statement at the start of the manuscript PDF file)?

The PLOS Data policy

Reviewer #1: Yes

Reviewer #2: Yes

**********

5. Is the manuscript presented in an intelligible fashion and written in standard English?

Reviewer #1: Yes

Reviewer #2: Yes

**********

**Reviewer #1** : General Comments:

The points raised in the previous review have been corrected. The manuscript has been improved and the modifications have been successfully integrated.

However, during the final verification of the methodology, a critical discrepancy was identified in relation to the Antimicrobial Susceptibility Testing (AST) guidelines, the specific content of the discs, and the choice of agents tested.

1-Major Comment: Inconsistency between Guidelines and Disc Concentration

In the Materials and Methods section, the authors note that the laboratory followed CLSI guidelines for interpreting the diameters of the inhibition zones of the disc diffusion tests. However, the specific concentration reported for two antimicrobial discs is not aligned with CLSI guidelines, but rather with EUCAST guidelines:

Piperacillin-tazobactam: The authors reported the use of a 30/6 µg disc. This is the EUCAST-specific concentration. CLSI guidelines require a significantly higher concentration of 100/10 µg for this antibiotic combination.

Ceftazidime (CAZ): The authors reported using a 10 µg disc, which is in accordance with EUCAST guidelines. CLSI guidelines require a 30 µg disc for the ceftazidime test.

Implication: Because the criteria for interpreting zone diameter depend strictly on the specific disc concentration, EUCAST disc data cannot be validly interpreted using CLSI cutoff point tables and vice versa. Using the wrong table for the wrong disc concentration invalidates the clinical categorization (Susceptible/Resistant) of the isolates. The authors should clarify which standard is used. If EUCAST discs were used, the dataset should be reanalyzed with EUCAST tables. If CLSI criteria were applied, these values are typographical errors that should be corrected to 100/10 µg and 30 µg.

2-Minor Comment: Omission of VRE in Study Limitations

Since vancomycin was not included in the test panel, the authors should address the epidemiological impact of this choice in the "Strengths and Limitations" section. The text needs to acknowledge that, without vancomycin data, it was impossible to determine the prevalence of vancomycin-resistant Enterococcus faecium (VRE). This data is important, as demonstrated by its position on the WHO's 2024 List of Priority Bacterial Pathogens. A brief sentence addressing this gap should be added to the limitations, and in Table 3, the value for vancomycin-resistant Enterococcus faecium should be "undetermined”. For carbapenem-resistant Pseudomonas aeruginosa, the value was zero, correct? It should be zero in the table.

3-Minor Comment: Conceptual Update on Pneumococcal Resistance

There is a conceptual discrepancy regarding surveillance priorities for Streptococcus pneumoniae. Since the WHO's 2017 list of priority pathogens—and maintained in the 2024 updates—the international focus for screening high-priority pneumococci has shifted from penicillin resistance to macrolide resistance. The authors should correct Table 3.

**Reviewer #2:**  There were only some scientific names that were not italicized on lines 48, 201,203,295, 299.

**********

what does this mean?). If published, this will include your full peer review and any attached files.

**Do you want your identity to be public for this peer review?**  If you choose “no”, your identity will remain anonymous but your review may still be made public.

For information about this choice, including consent withdrawal, please see our Privacy Policy

Reviewer #1: **Yes:** Clara Portugal

Reviewer #2: **Yes:** Merlyn Cruz

**********

[NOTE: If reviewer comments were submitted as an attachment file, they will be attached to this email and accessible via the submission site. Please log into your account, locate the manuscript record, and check for the action link "View Attachments". If this link does not appear, there are no attachment files.]

**Figure Resubmissions:**

# Author comment: Author response to Decision Letter 2

**DOI:** 10.1371/journal.pgph.0006705.r004

15 Jul 2026

Attachment Submitted filename: Response to ReviewersR2.docx

# Peer review: Decision Letter 2

**DOI:** 10.1371/journal.pgph.0006705.r005

**Authors:** Julia Robinson

5 Aug 2026

Bacterial spectrum and   proportion of   multidrug-resistant bacteria in clinical samples from a regional hospital in Lambaréné, Gabon (2017–2023): implications for antimicrobial resistance surveillance and public health

PGPH-D-26-01543R2

Dear Prof ADEGBITE,

We are pleased to inform you that your manuscript 'Bacterial spectrum and   proportion of   multidrug-resistant bacteria in clinical samples from a regional hospital in Lambaréné, Gabon (2017–2023): implications for antimicrobial resistance surveillance and public health' has been provisionally accepted for publication in PLOS Global Public Health.

Before your manuscript can be formally accepted you will need to complete some formatting changes, which you will receive in a follow up email. A member of our team will be in touch with a set of requests.

Please note that your manuscript will not be scheduled for publication until you have made the required changes, so a swift response is appreciated.

IMPORTANT: The editorial review process is now complete. PLOS will only permit corrections to spelling, formatting or significant scientific errors from this point onwards. Requests for major changes, or any which affect the scientific understanding of your work, will cause delays to the publication date of your manuscript.

If your institution or institutions have a press office, please notify them about your upcoming paper to help maximize its impact. If they'll be preparing press materials, please inform our press team as soon as possible -- no later than 48 hours after receiving the formal acceptance. Your manuscript will remain under strict press embargo until 2 pm Eastern Time on the date of publication. For more information, please contact globalpubhealth@plos.org.

Thank you again for supporting Open Access publishing; we are looking forward to publishing your work in PLOS Global Public Health.

Best regards,

Julia Robinson

Executive Editor

PLOS Global Public Health

***********************************************************

Reviewer Comments (if any, and for reference):

Reviewer's Responses to Questions

**Comments to the Author**

Reviewer #1: All comments have been addressed

Reviewer #2: All comments have been addressed

**********

publication criteria?

Reviewer #1: Yes

Reviewer #2: Yes

**********

3. Has the statistical analysis been performed appropriately and rigorously?

Reviewer #1: Yes

Reviewer #2: Yes

**********

4. Have the authors made all data underlying the findings in their manuscript fully available (please refer to the Data Availability Statement at the start of the manuscript PDF file)?

The PLOS Data policy

Reviewer #1: Yes

Reviewer #2: Yes

**********

5. Is the manuscript presented in an intelligible fashion and written in standard English?

Reviewer #1: Yes

Reviewer #2: Yes

**********

Reviewer #1: I would like to commend the authors for their revision efforts and for their work. All corrections have been made. I have no further comments and recommend the manuscript for publication

Reviewer #2: All the recommendations for the improvement and clarity of the manuscript have been addressed completely by the authors.

**********

what does this mean?). If published, this will include your full peer review and any attached files.

**Do you want your identity to be public for this peer review?**  If you choose “no”, your identity will remain anonymous but your review may still be made public.

For information about this choice, including consent withdrawal, please see our Privacy Policy

Reviewer #1: **Yes:** Clara Portugal

Reviewer #2: **Yes:** Dr. Merlyn C. Cruz

**********
